# Mechanisms of Sepsis-Induced Acute Lung Injury and Advancements of Natural Small Molecules in Its Treatment

**DOI:** 10.3390/ph17040472

**Published:** 2024-04-08

**Authors:** Yaxi Xu, Jianzeng Xin, Yupei Sun, Xuyan Wang, Lili Sun, Feng Zhao, Changshan Niu, Sheng Liu

**Affiliations:** 1 School of Pharmacy, Yantai University, Yantai 264005, China; unicy81@163.com (Y.X.); syp6935@163.com (Y.S.); 17851072916@163.com (X.W.); 2 School of Life Sciences, Yantai University, Yantai 264005, China; jianzeng77@sina.com; 3 College of Pharmacy, University of Utah, Salt Lake City, UT 84108, USA; lili.sun1989@gmail.com

**Keywords:** sepsis, ALI, natural small molecules, ARDS, sepsis therapy

## Abstract

Sepsis-induced acute lung injury (ALI), characterized by widespread lung dysfunction, is associated with significant morbidity and mortality due to the lack of effective pharmacological treatments available clinically. Small-molecule compounds derived from natural products represent an innovative source and have demonstrated therapeutic potential against sepsis-induced ALI. These natural small molecules may provide a promising alternative treatment option for sepsis-induced ALI. This review aims to summarize the pathogenesis of sepsis and potential therapeutic targets. It assembles critical updates (from 2014 to 2024) on natural small molecules with therapeutic potential against sepsis-induced ALI, detailing their sources, structures, effects, and mechanisms of action.

## 1. Introduction

Sepsis is an acute multi-organ dysfunction triggered by the host’s complex response to invasive pathogenic microorganisms [1]. This acute systemic inflammatory response to sepsis can precipitate a cascade of pathological and physiological alterations. Predominantly, the respiratory system, especially the lungs, is the first to be compromised, potentially leading to acute lung injury (ALI) [2]. Without prompt and effective treatment, this condition can progress into acute respiratory distress syndrome (ARDS), inflicting irreversible lung damage and potentially resulting in death [3]. ALI/ARDS is characterized by acute inflammation, disruption of endothelial barrier integrity, and alveolar epithelial damage, culminating in protein-rich interstitial edema and the infiltration of immune cells into the alveolar space [4,5]. Therefore, promoting lung repair and mitigating lung inflammation are potential therapeutic strategies for ALI/ARDS [6].

The conventional treatment for sepsis-induced ALI/ARDS encompasses antibiotic therapy, fluid resuscitation, blood product transfusion, and the administration of vasoactive drugs. Antibiotic therapy is crucial for addressing the underlying infection. Fluid resuscitation is employed to enhance tissue perfusion and ensure blood pressure stability. Transfusing blood products helps maintain an adequate oxygen supply to the tissues, while vasoactive drugs adjust vascular tone and support consistent blood pressure levels [7].

Conventional treatments, however, come with several limitations. Firstly, the overuse of antibiotics may contribute to the emergence of bacterial resistance. Second, fluid resuscitation can lead to fluid overload and pulmonary edema. Blood product transfusion carries the risk of transfusion-related complications. Lastly, the administration of vasoactive drugs can result in significant blood pressure fluctuations and elevate the risk of cardiovascular complications [2,7,8].

To address these challenges, there has been a call for further research and the development of new therapeutic strategies to enhance treatment efficacy and minimize complications. These strategies include advances in immunomodulatory, cellular, and anti-inflammatory therapy [9]. Recent clinical research has highlighted the promising efficacy of natural small molecules in treating sepsis-induced lung injury, offering new avenues for treatment [10]. Natural small molecules provide several significant advantages as therapeutic agents for sepsis-induced ALI. Firstly, natural compounds are frequently associated with fewer side effects compared to synthetic drugs due to their compatibility with biological systems. Secondly, they provide a rich source of novel bioactive compounds, many of which have been historically utilized in traditional medicine and are now recognized for their potent anti-inflammatory, antioxidant, and immunomodulatory properties. These characteristics are especially relevant in the context of sepsis.

Furthermore, natural small molecules can offer multifaceted therapeutic effects, targeting multiple pathways involved in sepsis-induced ALI [9,10,11]. These advantages make natural small molecules a promising alternative treatment option for sepsis-induced ALI. This review aims to summarize the pathogenesis of sepsis and potential therapeutic targets and further delves into the latest developments in natural small molecules for treating sepsis-induced ALI.

## 2. Pathogenesis of Sepsis-Induced ALI and Potential Therapeutic Targets

In the pathological process of sepsis, the lungs are the most severely affected organ, leading to ALI [12]. Initially, the recruitment of inflammatory cells increases epithelial and endothelial permeability, along with the leakage of proteins and cellular contents. The cascade culminates in interstitial inflammation marked by heightened oxidative stress, lung barrier dysfunction, pulmonary edema, and vascular leakage. The etiological diagnosis of the disease is linked to an uncontrolled and complex interplay of inflammatory cytokines and cellular mediators, resulting in injury to the alveolar-capillary unit [13]. Then, ALI can evolve into ARDS, a highly heterogeneous and life-threatening severe form of acute respiratory failure, which is characterized by acute episodes of hypoxemia and bilateral pulmonary infiltrates [14,15].

**Figure 1:**
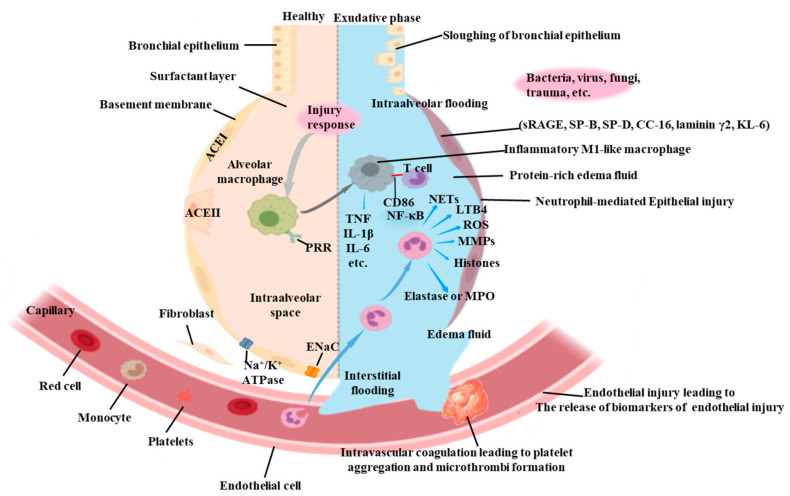
Pathological comparison of ALI induced by sepsis. During the exudative phase, inflammatory cells release inflammatory mediators, accumulating in the alveoli and capillaries, which results in the damage of the alveolar wall and the increasing of permeability, further leading the seepage of fluid, proteins, and inflammatory cells into the alveolar space.

Sepsis-induced ALI is a common and life-threatening condition among critically ill patients, with a highly complex pathogenesis [16]. Specifically, the mechanism behind sepsis-associated ALI encompasses multiple factors, including inflammatory response, oxidative stress, inflammatory cell infiltration, and vascular leakage (see Figure 1). Initially, a systemic inflammatory response triggers the infiltration of lung tissues by inflammatory cells and the release of inflammatory mediators such as tumor necrosis factor-α (TNF-α), interleukin-1β (IL-1β), and interleukin-6 (IL-6), which further activate key inflammatory signaling pathways, including nuclear factor-κB (NF-κB) and the phosphatidylinositol-3 kinase (PI3K)/protein kinase B (AKT) pathway, further aggravating lung injury [16,17]. Concurrently, sepsis-induced inflammation generates oxidative stress, which may increase free radicals and oxidized substances within cells. Oxidative damage leads to cell membranes’ lipid peroxidation and oxidized proteins’ accumulation, intensifying lung injury [12,14,18].

**Figure 2:**
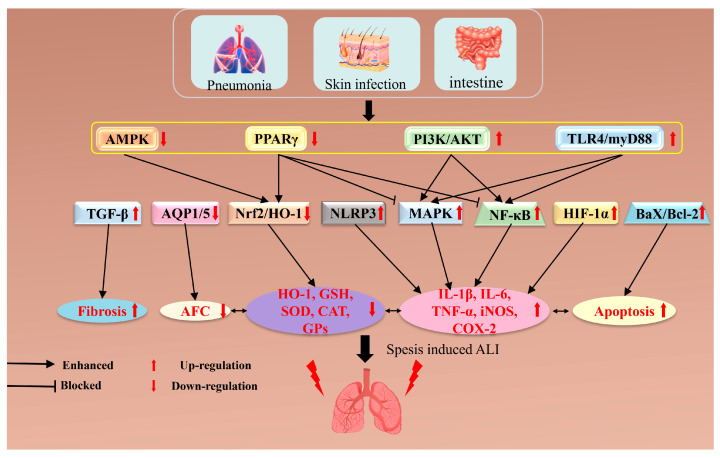
The mechanisms or pathways associated with sepsis-induced ALI. Uncontrolled infectious inflammation in areas such as the lungs, skin, or small intestine can precipitate sepsis, which, in turn, may lead to varying degrees of tissue and organ loss, including lung injury. One significant pathway affected is the AMPK/PPARγ pathway; its down-regulation leads to the decreased activation of Nrf2, further diminishing the antioxidant response. Consequently, this affects the expression of antioxidant enzymes such as SOD and CAT, impairing the ability to scavenge free radicals. The compromised state of the AMPK/PPARγ pathway in sepsis renders cells more vulnerable to oxidative damage and inflammatory responses due to insufficient antioxidant defense capabilities. Additionally, the activation of the PI3K/TLR4 pathway facilitates the activation of NF-κB, resulting in the up-regulation of inflammation-related genes. NF-κB plays a crucial role in the regulation of the inflammatory response. Concurrently, activating the PI3K/TLR4 signaling pathway stimulates the MAPK pathway, including JNK, p38, and ERK. These pathways elevate inflammatory mediators like TNF-α and iNOS, further exacerbating the inflammatory response.

Moreover, the inflammatory response promotes extensive infiltration of inflammatory cells, such as neutrophils and monocytes, into lung tissue. These cells release inflammatory mediators and proteases, contributing to the destruction of alveolar walls and the development of pulmonary edema. Lastly, an elevated level of inflammatory mediators enhances the permeability of the pulmonary vasculature, allowing plasma and inflammatory cells to leak into the interstitial and alveolar spaces, further worsening lung injury [19]. In summary, the pathogenesis of ALI in sepsis involves multiple interconnected pathways, as illustrated in Figure 2 [20].

### 2.1. Mitogen-Activated Protein Kinase Pathway (MNK)

The MNK kinase family comprises two distinct members, MNK1 and MNK2, identified as serine/threonine kinases. They were initially discovered as substrates for extracellular signal-regulated kinases (Erk1 and Erk2) by two independent screens [21]. Owing to their capacity to respond to external stimuli, the MNK kinase family has been implicated in various biological functions [22]. Recent research has highlighted the mitogen-activated protein kinase (MAPK) pathway in anti-inflammation and prevention of tissue damage [23].

Tang’s group [24] conducted further studies, revealing that targeting MNKs to inhibit the MAPK pathway could effectively prevent and treat ALI. Experiments involving model mice treated with MNK1 and MNK2 inhibitors showed a significant reduction in inflammatory indices triggered by lipopolysaccharide (LPS). Additionally, the involvement of MNK2 in the lung injury process was demonstrated through MNK2 knockout mice experiments. These findings suggest that MNK inhibition has a definitive therapeutic effect on ALI mice. Specifically, the role of infection-induced lung inflammation and injury is pivotal, indicating that developing MNK-specific inhibitors could advance drug development for ALI and offer new therapeutic strategies for its treatment [23,24].

### 2.2. Rho-Associated Protein Kinase Pathway (ROCK)

ROCK, comprising two isozymes, ROCK1 and ROCK2, belongs to the serine/threonine kinase family.The role of the ROCK signaling pathway in the pathophysiology of ALI and the relevance of specific Rho-kinase inhibitors in its prevention and treatment are of particular interest. This pathway contributes to endothelial barrier dysfunction and edema, which are the hallmark features of ALI. In recent years, there has been a surge in research focusing on Rho-kinase inhibitors, driven by the hypothesis that they have therapeutic effects on lung diseases [25].

Karimi’s group [26] studied different Rho kinase inhibitors to evaluate their therapeutic effects on ALI of various causes. Preliminary preclinical studies have shown that Rho-related signaling pathways play an essential role in the pathogenesis of ALI, and Rho kinase inhibitors have good potential in alleviating the disease of ALI and can bring considerable benefits. However, as a new therapeutic approach, Rho kinase inhibitors need to be further verified by advanced clinical trials.

### 2.3. Myeloid Differentiation Factor 2 Pathway (MD2)

MD2, known as myeloid differentiation protein 2, plays a pivotal role in the immune system by interacting with Toll-like receptor 4 (TLR4) to regulate immune and inflammatory responses. By blocking the interaction between MD2 and LPS, the inflammatory response can be inhibited, thereby reducing the symptoms of inflammatory diseases [27]. Liang’s research group [28] has made significant progress. They have reported a series of synthetic compounds that have protective effects on septic shock and lung inflammation in vivo. These compounds effectively hinder the binding of LPS to MD2 and show anti-inflammatory properties in LPS-stimulated macrophages. This study provides a new therapeutic approach for the control and treatment of ALI.

### 2.4. Toll-like Receptors Pathway (TLRs)

Toll-like receptors (TLRs) are a pivotal class of protein receptors within the human immune system, designed to recognize and bind to molecular patterns exhibited by pathogens, thus initiating an immune response. The primary function of TLRs is to galvanize the immune system and spark an inflammatory response that facilitates a defense against pathogens [29,30].

Fan [31] discussed the internal mechanism of ALI, and their results emphasized the reasons that the vascular endothelial cells activate the reactive oxygen species (ROS) in alveolar macrophages produced after trauma, leading to inflammatory response and subsequent lung injury. TLRs can recognize pathogen-associated molecular patterns (PAMPs) and damage-associated molecular patterns (DAMPs) and are considered critical factors in the development of ALI after trauma. Consequently, TLRs, together with ROS, have become potential therapeutic targets for controlling post-traumatic ALI, emphasizing the importance of further research in this field to develop effective treatments.

In 2018, Liu [32] carried out a study focusing on the activation and role of TLRs in sepsis-induced ALI. The results of this study further demonstrated that the silencing of Toll-like receptor 2 (TLR2), TLR4, or Toll-like receptor 9 (TLR9) can reduce the infiltration of immune cells, thereby reducing lung injury. Meanwhile, it also proved that various TLRs are overexpressed and involved in the inflammatory response in the sepsis-induced ALI model.

Myeloid differentiation major response 88 (MyD88), as a critical signaling molecule in the immune system, acts as an essential adaptor in the downstream signaling pathways of the TLR and interleukin-1 receptor (IL-1R) families [33].

In 2021, a study conducted by Cai [34] underscored the significance of crafting targeted therapies for ALI, focusing on specific elements within the immune response pathway. TLRs emerge as critical targets in addressing sepsis-induced ALI, with MyD88 playing a pivotal role in the signaling cascade. The study suggests that TLRs and MyD88 could be instrumental in devising practical treatment approaches for this condition.

### 2.5. NADPH Oxidase (NOX)

NOX enzymes, located within cell membranes, play a critical role in catalyzing the intracellular oxidation of NADPH, which leads to the generation of ROS, such as superoxide anion (O^2−^). These enzymes are central to various physiological and pathological processes, including cellular signaling, immune responses, and oxidative stress. However, excessive activation of NOX can result in cellular damage and contribute to disease development. Pache [35] explored the pathogenesis of ALI and ARDS. Their data showed that NOX4 inhibitors could significantly reduce Transforming growth factor-beta(TGF-β1)-induced epithelial cell death and fibroblast differentiation. Since NOX4 acts upstream of the adverse effects of TGF-β1, targeting NOX4 can more effectively prevent TGF-β1-driven harmful cascade reactions, making NOX4 inhibitors a potential method for treating ALI.

Cai’s teams [36] studied the effect of oxidative stress on sepsis-induced ARDS. In cultured human pulmonary microvascular endothelial cells, inhibition of NOX4 reduced LPS-induced ROS production, Ca^2+^/calmodulin-dependent protein kinase II(CaMKII)/ERK1/2/myocin light chain kinase (MLCK) activation, and endothelial cell barrier function damage. As a result, targeting NOX4 is a novel and promising method for treating ALI.

### 2.6. Adenosine Monophosphate-Activated Protein Kinase Pathway (AMPK)

AMP-activated protein kinase (AMPK) is a critical enzyme that is an energy sensor and regulator of cell metabolic homeostasis. It is activated to facilitate energy production and conservation in response to the decreased cellular energy levels. AMPK orchestrates the regulation of various metabolic pathways through the phosphorylation of multiple targets to influence glucose and fatty acid metabolism, protein synthesis and degradation, cell proliferation, and apoptosis. Consequently, AMPK is pivotal in maintaining cellular energy balance and metabolic health [37].

In a notable study from 2019, Zingarelli [38] demonstrated the potential of AMPK activation in alleviating sepsis-induced ALI in adult mice. The study further explored the mechanism of A769662 and revealed its regulatory effect on the AMPK signaling pathway. In addition, A769662 was found to enhance mitochondrial biogenesis and may contribute to lung recovery by activating the nuclear metabolic regulator peroxisome proliferator-activated receptor-γ coactivator 1-α (PGC-1α). Therefore, the results suggest that AMPK can be used as a feasible therapeutic target for treating sepsis-induced ALI.

### 2.7. NF-κB Pathway

NF-κB, a family of inducible transcription factors, plays crucial regulatory roles in immune and inflammatory responses. NF-κB proteins transition from the cytoplasm to the nucleus by activating a sequence of signaling cascades, which stimulates the expression of specific genes. This activation is linked to the development of numerous diseases, such as chronic inflammatory diseases, asthma, rheumatoid arthritis, and inflammatory bowel disease [39].

Rahman [40] explores the potential of therapeutically targeting NF-κB in ALI; it highlights the significance of autophagy in ALI and mentions the role of the mammalian target of rapamycin (mTOR) signaling pathway in the regulation of kappa B kinase (IKK)/NF-κB. Given these insights, NF-κB emerges as a promising target for treating sepsis-induced ALI. Inhibiting NF-κB activation could mitigate the inflammatory response and tissue damage associated with ALI. Several small molecule inhibitors have been developed to curb NF-κB activation by targeting various components of NF-κB signaling pathway.

In 2022, Yang shed light on the impact of Shionone [41] on sepsis-induced ALI and its mechanism of action via the extracellular matrix protein 1 (ECM1)/signal transducer and activator of transcription 5 (STAT5)/NF-κB pathway. The study revealed that Shionone mitigated sepsis-induced lung damage by influencing the ECM1/STAT5/NF-κB pathway. Furthermore, Shionone was shown to alleviate sepsis-induced lung injury by suppressing the inflammatory response, reducing lung edema and the infiltration of inflammatory cells.

However, existing pharmacological interventions show that targeting NF-κB has demonstrated variable outcomes in clinical trials, possibly due to NF-κB’s protective role in resolving inflammation and injury. Thus, the modulation of NF-κB remains crucial for effectively treating ALI. A nuanced approach is needed to harness its therapeutic potential without disrupting its beneficial effects.

### 2.8. PI3K Pathway

PI3K, an enzyme pivotal in regulating many cellular processes, including cell survival, cycle progression, differentiation, senescence, and metabolism, plays a crucial role in intracellular signaling pathways [42]. During inflammatory responses, injury, and infection, its activation influences essential events, including immune cell activation, inflammatory response regulation, and fibrosis development. Thus, targeting the PI3K pathway offers a promising approach for treating diseases associated with infection, inflammation, and fibrosis [43].

Hirsch [43,44] and Zhu [45,46] proved that inhibition of the PI3K signaling pathway can inhibit inflammatory response and reduce the activation of immune cells, thereby reducing polar lung injury caused by sepsis. Although PI3K inhibitors have been clinically tested in other diseases, such as pneumonia and pulmonary fibrosis, and have shown specific efficacy, the safety and efficacy of PI3K inhibitors in treating sepsis-induced polar lung injury still need to be further evaluated.

### 2.9. Nucleotide-Binding Oligomerization Domain-like Receptor Protein 3 Pathway (NLRP3)

NLRP3, an inflammation-associated protein, is a critical intracellular receptor predominantly in immune cells. It plays a pivotal role in inflammation and immune responses [47].

Zhang’s group [48] identified NLRP3 as a potential therapeutic target for sepsis-induced ALI. Their research focused on the protective effects of tangeretin (TAN) on sepsis-induced ALI and its regulatory mechanism on NLRP3 inflammasomes. The findings revealed that TAN mitigated sepsis-induced ALI in mice through various pathways, primarily by inhibiting ROS-mediated activation of the NLRP3 inflammasome and modulating the polo-like kinase 1 (PLK1)/AMPK/dynamin-related protein 1(DRP1) signaling pathway.

In a subsequent study in 2023, Pan explored the protective effects of colchicine against LPS-induced lung injury and its underlying mechanisms. It was discovered that colchicine inhibits the phosphorylation of the signal transducer and activator of transcription 3 (STAT3), which regulates the activation of NLRP3, thereby reducing the inflammatory response and apoptosis [47,48].

### 2.10. Other Potential Therapeutic Targets

In addition, clinical studies have found that sepsis-induced ALI can also be significantly attenuated through the effects of specific protein molecules including phospholipase A2 type IIA (Pla2g2a)-epidermal growth factor receptor (EGFR) [49], estrogen-related receptor alpha (ERRα) [50,51], mucin 1 (MUC1) [52], microRNA 199a (miR-199a) [53,54], gasdermin D (GSDMD) [55,56], tyrosine kinase with immunoglobulin, and epidermal growth factor homology domains 2(Tie2) [57,58]. These findings open new avenues for the development of therapeutic strategies for sepsis-induced ALI.

## 3. Natural Small Molecules Used for the Treatment of Sepsis-Induced ALI

Natural small molecules are pharmacologically active compounds with biological activity or nutritional value and low molecular weight. They are derived or synthesized from plants, animals, or microorganisms of natural origin. Once developed into drugs, these natural small molecules often exhibit superior bioavailability, pharmacokinetic properties, and pharmacodynamic activity [59].

Natural small molecule drugs typically target multiple aspects of sepsis pathogenesis simultaneously, such as inflammatory response, oxidative stress, and immune regulation, thereby enhancing therapeutic efficacy. Moreover, compared to synthetic drugs, natural small-molecule drugs, predominantly sourced from natural entities like plants, animals, or microorganisms, tend to have fewer toxic side effects, rendering them safer and more reliable for treating sepsis [59,60]. Traditional antibiotic treatments may fall short, particularly in sepsis, where patients frequently face bacterial resistance. With their diverse antibacterial mechanisms, natural small-molecule drugs can effectively combat bacterial resistance and enhance treatment outcomes [60]. Furthermore, these drugs have the potential to modulate immune system functions, bolster the body’s defenses, mitigate inflammatory responses, and facilitate recovery.

Additionally, the diverse origins of natural small-molecule drugs from plants, animals, or microorganisms ensure ample supply [61]. Most notably, the production process of these drugs is relatively simple and sustainable, favoring large-scale production and widespread application. In conclusion, with their multifaceted benefits in sepsis treatment, natural small-molecule drugs represent a promising therapeutic avenue [62]. The following compounds (**1**–**32**) are selected natural products reported with promising anti-sepsis-induced ALI properties. For detailed compound information, including plant source, compound classification, and chemical structure, please see Table 1, Table 2, Table 3, Table 4 and Table 5.

### 3.1. Dihydromyricetin (DHM, ***1***)

DHM (Table 1), a compound extracted from the young stems and leaves of the grapevine plant, boasts a variety of pharmacological effects, including anti-inflammatory, antioxidant, and anticancer properties [63]. Studies have demonstrated that DHM can mitigate sepsis-induced ALI by inhibiting apoptosis dependent on the NLRP3 inflammasome. In sepsis, the activation of the NLRP3 inflammasome escalates apoptosis and the inflammatory response, damaging lung tissues.

DHM counteracts the activation of the NLRP3 inflammasome by stimulating the nuclear factor erythroid 2-related factor 2 (Nrf2) signaling pathway, which diminishes both apoptosis and the inflammatory response, thereby safeguarding lung tissues from damage. Specifically, DHM prevents the formation of NLRP3 inflammasomes and the assembly of ASC (adapter protein), effectively inhibiting the activation of inflammatory vesicles. Furthermore, DHM enhances the activity of Nrf2, fostering antioxidant responses that mitigate damage from oxidative stress and inflammation. In summary, DHM offers a therapeutic benefit in treating sepsis-induced ALI by blocking NLRP3 inflammasome-dependent apoptosis and alleviating inflammatory responses and oxidative stress [64].

### 3.2. Myricetin (***2***)

Myricetin (Table 1), a natural flavonoid primarily found in plants *Myrica rubra*, is known for its anti-inflammatory and antioxidant properties. Its pharmacological effects have been well-documented across numerous studies [65].

Myricetin is considered to have a potential pharmacotherapeutic role in ameliorating sepsis-associated ALI. The mechanism by which Myricetin acts in sepsis-induced ALI is to exert a protective effect by modulating the Nrf2/ heme oxygen-ase-1(HO-1) pathway. Myricetin significantly improved survival in a mouse model of sepsis and attenuated pathologic changes, inflammatory responses, oxidative stress, and mitochondrial damage. Additionally, Myricetin up-regulated the expression levels of the transcription factors Nrf2 and HO-1 and increased the DNA-binding activity of Nrf2. The Nrf2/HO-1 pathway is an influential anti-inflammatory, and its modulation attenuates sepsis-induced lung injury.

Further research showed that the protective effect of Myricetin on sepsis-induced lung injury was found to be dependent on the presence of Nrf2 through the establishment of an Nrf2 knockout mouse model. The activation of Nrf2 could contribute to the high expression of genes, such as HO-1, which protects the organism from sepsis. In summary, Myricetin is protective by regulating the Nrf2/HO-1 pathway and attenuating sepsis-induced ALI [65,66].

### 3.3. Luteolin (***3***)

Luteolin (Table 1), a naturally occurring flavonoid compound found in various plants, displays a broad spectrum of pharmacological effects, including antioxidant, anti-inflammatory, and neuroprotective properties. It is present in numerous foods, such as carrots, chili peppers, celery, olive oil, peppermint, and green tea. The chemical structure of luteolin includes two benzene rings and an oxygenated heterocyclic ring adorned with multiple hydroxyl functional groups and a C2–C3 double bond, highlighting its potential medicinal value [67].

Studies indicate that luteolin can ameliorate sepsis-induced ALI through several mechanisms. Primarily, luteolin can reduce pulmonary edema and protein leakage, as evidenced by experiments where pretreatment with luteolin lessened both edema and protein content in lung tissue and bronchoalveolar lavage fluid. Additionally, luteolin can suppress the inflammatory response, notably by reducing the expression of intercellular adhesion molecule (ICAM)-1 and NF-κB. Pretreatment with luteolin decreased the mRNA expression of ICAM-1 and the activation of NF-κB. Moreover, luteolin offers therapeutic benefits by mitigating oxidative stress and partly inhibiting the inducible nitric oxide synthase (iNOS) pathway. It was observed that luteolin pretreatment lowered lipid peroxidation and enhanced the activity of antioxidant enzymes such as superoxide dismutase (SOD) and catalase (CAT) in lung tissues. In summary, luteolin delivers its therapeutic effects in combating sepsis-induced ALI through diverse pathways, including damping down inflammatory responses, alleviating pulmonary edema, reducing the expression of ICAM-1 and NF-κB, lessening oxidative stress, and partially blocking the iNOS pathway. These findings underscore luteolin’s potential as a promising agent in treating sepsis-induced ALI [68].

### 3.4. Quercetin (***4***)

Quercetin (Table 1), a flavonoid and secondary plant metabolite, is a natural pigment found abundantly in fruits, vegetables, nuts, wines, and seeds. It is also available as a dietary supplement. This compound is renowned for its wide range of biological activities and pharmacological benefits, including its antioxidant, antibacterial, antiviral, free radical scavenging, gastric mucosal protective, and immune-function-modulating properties [69].

In sepsis-induced ALI, quercetin has been shown to suppress the release of inflammatory mediators such as TNF-α, IL-1β, and IL-6 [70]. These mediators are pivotal in regulating inflammation during sepsis, and quercetin’s anti-inflammatory effects can significantly mitigate the inflammatory response, thus reducing the severity of lung damage. Furthermore, sepsis-induced ALI frequently leads to escalated oxidative stress, characterized by an increase in free radicals and oxidative harm. Quercetin’s potent antioxidant capability enables it to neutralize free radicals, alleviate oxidative stress, and safeguard lung tissues against oxidative injury. Sepsis-induced ALI also triggers vascular endothelial dysfunction, increasing vascular permeability and leading to pulmonary edema, while abnormally activating the immune system and causing an excessive inflammatory response. Quercetin addresses sepsis-induced ALI through various mechanisms, including anti-inflammatory and antioxidant effects, protection of vascular endothelial function, and immune response modulation. Nevertheless, additional research is required to confirm its effectiveness and identify the optimal dosage and timing for therapy.

### 3.5. Baicalein (***5***)

Baicalein (Table 1), a flavonoid primarily found in plants such as *Scutellaria baicalensis*-Georgi, exhibits a spectrum of pharmacological properties, including anti-inflammatory, antioxidant, antiviral, and antitumor activities [71]. This compound is effective in mitigating sepsis-induced ALI through the inhibition of the TLR4 signaling pathway. Baicalein explicitly prevents the binding of LPS to MD2, blocking the formation of the TLR4-MD2 signaling complex and consequently inhibiting the LPS-induced inflammatory response. Research involving a mouse model has demonstrated that Baicalein significantly reduces inflammatory cell infiltration in lung tissues and decreases the presence of inflammatory cells. Moreover, it curtails the production of pro-inflammatory factors released by these cells, such as IL-6 and TNF-α, further alleviating sepsis-induced ALI. In summary, Baicalein offers a therapeutic benefit in sepsis-induced ALI by disrupting the TLR4-MD2 signaling pathway and reducing the inflammatory response and the infiltration of inflammatory cells [72].

### 3.6. Kaempferol (***6***)

Kaempferol (Table 1) is a flavonoid compound found in many foods and traditional herbs. It has numerous pharmacological effects, including antioxidant, anti-inflammatory, antimicrobial, anticancer, cardioprotective, neuroprotective, hypoglycemic, and antiallergic effects [73].

In sepsis-induced ALI, Kaempferol demonstrates significant therapeutic benefits. Pretreatment with Kaempferol notably diminishes the water content in the lungs, effectively reducing pulmonary edema. This pretreatment also lowers the levels of pro-inflammatory cytokines (e.g., IL-6, IL-1β, and TNF-α) in the plasma and lung tissues, thereby mitigating the inflammatory response. Furthermore, it boosts the activity of antioxidant enzymes (such as SOD and catalase) and increases the presence of non-enzymatic antioxidant substances (like reduced glutathione), reducing oxidative stress. Kaempferol’s ability to decrease nitric oxide (NO) levels in lung tissues and suppress the expression of inducible nitric oxide synthase (iNOS) further curtails NOX production. Additionally, it down-regulates the mRNA expression level of ICAM-1 and lowers the expression of cell adhesion molecules. In summary, Kaempferol provides therapeutic effects on ALI triggered by sepsis through multiple mechanisms: it decreases pulmonary edema, inhibits the inflammatory response, enhances antioxidant defenses, and suppresses the activity of iNOS and ICAM-1 [74].

### 3.7. Cardamonin (***7***)

Cardamonin (Table 1) is a natural flavonoid derived from cardamom, which has demonstrated anti-inflammatory, antibacterial, and antidiabetic capabilities in various studies. This compound is noted for its diverse biological activities, including antioxidant, anti-inflammatory, antitumor, and cell cycle modulation effects [75].

Research findings reveal that Cardamonin can obstruct the production of inflammatory cytokines induced by LPS by binding to MD2. Furthermore, it inhibits the activation of the NF-κB and c-JunN-terminal kinases (JNK) signaling pathways, reducing inflammatory cytokine production. In vivo studies have shown that Cardamonin treatment mitigates LPS-induced lung injury, decreases the production of systemic inflammatory cytokines, and reduces macrophage infiltration in the lungs. Additionally, Cardamonin treatment has been observed to enhance the survival rates of septic mice. Moreover, by interacting with MD2, Cardamonin inhibits the activation of the TLR4/MD2-MyD88-MAPK/NF-κB signaling pathway, thereby alleviating the impact of sepsis-induced ALI [76].

### 3.8. Isoliquiritigenin (ISL, ***8***)

Isoliquiritigenin (ISL) (Table 1) is a bioactive compound extracted from the roots of licorice-containing plants, *Glycyrrhiza glabra* and *Glycyrrhiza mongolica*. It is found in common foods and traditional medicines, with its derivative ISL utilized in food additives and various disease treatments, such as anticancer and antibiotic therapies [77].

ISL has various pharmacological activities, including anti-inflammatory, antimicrobial, antioxidant, anticancer, immunomodulatory, hepatoprotective, and cardioprotective properties. As a flavonoid with anti-inflammatory capabilities, research has demonstrated ISL’s potential to provide therapeutic benefits for sepsis-induced ALI by dampening inflammatory responses. Specifically, ISL has been shown to suppress the expression of inflammatory markers in LPS-stimulated murine peritoneal macrophages. In a mouse model of ALI, ISL effectively prevented LPS-induced structural harm and infiltration by inflammatory cells. Furthermore, ISL pretreatment mitigated sepsis-induced damage to the lungs and liver, alongside a notable decrease in inflammatory reactions. These protective outcomes are achieved ex vivo through the blockade of the inflammatory response via the NF-κB pathway. Consequently, ISL emerges as a promising therapeutic candidate for managing sepsis-induced ALI [78,79].

### 3.9. Afzelechin (***9***)

Afzelechin (Table 1), a natural compound within the flavonoid family, is derived from the *Bergenia ligulata* plant. This compound exhibits various pharmacological effects, including antioxidant, anticancer, antimicrobial, and cardiovascular benefits. It mitigates the inflammatory response and protects the lungs from damage by modulating cytokine levels and related signaling pathways. Afzelechin is a promising candidate for the research and development of treatments targeting sepsis-induced ALI. Experimental results demonstrate that it effectively reduces inflammatory cytokine levels in serum and lung tissues, thus protecting against LPS-induced lung injury. The study suggests that Afzelechin alleviates sepsis-induced ALI by controlling the cytokine storm and suppressing inflammation by modulating TLR4/NF-κB, PI3K/Akt, Hippo, and Rho signaling pathways. This study establishes a foundation for further investigation into the mechanisms of Afzelechin and its potential as a therapeutic option for sepsis-induced ALI [80].

### 3.10. Calycosin (***10***)

Calycosin (Table 1), a distinctive phytoestrogen found in the Astragalus root, showcases an array of pharmacological activities such as anticancer, anti-inflammatory, anti-osteoporotic, neuroprotective, and hepatoprotective effects. It operates through various signaling pathways, including PI3K/Akt/mTOR, WDR7-7-GPR30, and Rab27B-β-catenin-VEGF, exhibiting diverse pharmacological impacts [81].

Calycosin’s therapeutic efficacy against sepsis-induced ALI involves inhibiting the high mobility group protein 1(HMGB1)/MyD88/NF-κB pathway. HMGB1 is a non-histone nuclear protein widely found in cells, which plays a pro-inflammatory role in sepsis. Calycosin can inhibit the release and activation of HMGB1, thereby attenuating the inflammatory response. Additionally, calycosin can inhibit the activation of MyD88 and NF-κB, further reducing the production of inflammatory mediators. NLRP3 is a complex present in the cytoplasm that regulates inflammatory response. Calycosin can inhibit the activation of NLRP3 inflammatory vesicles and reduce the release of inflammatory mediators. ALI caused by sepsis is often accompanied by pulmonary edema and apoptosis. Calycosin can reduce pulmonary edema and lower the wet or dry weight ratio of lung tissue, improving lung function. In addition, calycosin can reduce apoptosis and protect lung tissue from injury. Calycosin has antioxidant and anti-inflammatory effects, which can reduce the damage of oxidative stress and inflammation in sepsis-induced ALI. Calycosin can also alleviate sepsis-induced ALI by inhibiting mitochondrial ROS-mediated activation of inflammatory vesicles [82].

It has been found that sepsis and lipopolysaccharide (LPS) injections lead to increased production of IL-1β and IL-18 in lung tissues, along with activation of NLRP3 inflammatory vesicles and cysteine-requiring aspartate protease-1 (caspase-1). Calycosin treatment significantly reduces IL-1β and IL-18 levels and inhibits NLRP3 inflammasome activation and caspase-1 activity. Furthermore, calycosin attenuates oxidative stress in lung tissue, decreases malondialdehyde (MDA) levels, and increases superoxide dismutase (SOD) and glutathione (GSH) levels. Overall, calycosin exerts therapeutic effects on sepsis-induced ALI by inhibiting inflammatory vesicle activation and attenuating oxidative stress [83].

### 3.11. Astilbin (***11***)

Astilbin (Table 1), a flavonoid compound predominantly found in plants like *Hypericum perforatum*, exhibits a broad spectrum of pharmacological effects, including anti-inflammatory, antioxidant, anticancer, and hepatoprotective properties. It effectively inhibits inflammatory reactions, alleviating pain and swelling associated with inflammation. Astilbin is a potent antioxidant that neutralizes free radicals, minimizing cellular damage from oxidative stress. Additionally, it protects the liver by reducing liver injury and fibrosis and fostering the repair and regeneration of liver cells. Astilbin is a multifaceted compound with significant potential therapeutic benefits for inflammation, oxidative stress, cancer, and liver diseases [84].

Astilbin can also attenuate sepsis-induced ALI by inhibiting the expression of macrophage inhibitory factor (MIF). This cytokine is crucial in regulating inflammatory and immune responses during sepsis. It has been found that overexpression of MIF exacerbates inflammatory response and increases tissue damage. Astilbin, conversely, can inhibit the expression and production of MIF, thereby attenuating sepsis-induced ALI. Moreover, Astilbin reduces sepsis-induced pulmonary edema and inflammatory cell infiltration. Lung edema is one of the main features of ALI, while inflammatory cell infiltration manifests the inflammatory response. Treatment with Astilbin can perceptibly decrease lung edema and inflammatory cell infiltration, thus improving the pathological changes of sepsis-induced ALI. In summary, Astilbin attenuates sepsis-induced ALI by inhibiting the expression and production of MIF [85].

### 3.12. Gossypin (***12***)

Gossypin (Table 1), a flavonoid compound predominantly found in the petals and buds of cotton plants used in traditional Chinese medicine [86], exhibits a broad spectrum of pharmacological effects, including antioxidant, anti-inflammatory, analgesic, antitumor, anticancer, and antidiabetic properties. Studies, including those by Song, have highlighted Gossypin’s potent antioxidant capability, which neutralizes free radicals and diminishes cellular damage caused by oxidative stress. Furthermore, its anti-inflammatory properties effectively suppress the inflammatory response by inhibiting the release of inflammatory mediators. Gossypin has diverse pharmacological effects, offering extensive therapeutic potential across various applications [87].

### 3.13. Isoorientin (***13***)

Isoorientin (Table 1), a C-glucosyl flavonoid found in a variety of plants such as rooibos, wheat tea, and several herbs, exhibits a broad spectrum of pharmacological effects, including antioxidant, anti-inflammatory, antidiabetic, and anti-obesity properties. Studies have demonstrated that Isoorientin can improve metabolic disorders by mitigating oxidative stress and inflammatory responses [88].

Isoorientin reduces lung tissue inflammation by decreasing inflammatory cell infiltration and releasing inflammatory mediators. Given that sepsis-induced ALI is often marked by an increase in oxidative stress, Isoorientin’s antioxidant properties help to lower the production of oxygen free radicals and enhance the activity of antioxidant enzymes, thereby diminishing oxidative damage to lung tissues. Additionally, Isoorientin has been found to decrease the expression of vascular endothelial cell adhesion molecules, reducing the adhesion and migration of inflammatory cells to vascular endothelium and alleviating lung tissue inflammation. Furthermore, Isoorientin’s therapeutic impact is bolstered by activating the janus kinase 2 (JAK2)/STAT3 signaling pathway. This activation reduces the shedding of the endothelial protein C receptor (EPCR) on the endothelial cell membrane and stimulates the JAK2/STAT3 pathway, further inhibiting inflammatory responses and oxidative stress. In summary, through mechanisms such as reducing lung inflammation, enhancing antioxidant defenses, inhibiting the adhesion and migration of inflammatory cells, and activating the JAK2/STAT3 pathway, Isoorientin presents a compelling therapeutic option for managing sepsis-induced ALI. These insights lay a theoretical foundation for considering Isoorientin as a potential medication for treating sepsis-induced ALI [89].

### 3.14. Silymarin (***14***)

Silymarin (Table 1), derived from milk thistle (*Silybum marianum*), is an extract celebrated for centuries as a herbal remedy primarily for liver ailments. This compound plays a crucial role in mitigating the adverse effects of sepsis by counteracting the excessive production of reactive oxygen species, which triggers oxidative stress, leading to cellular and tissue damage. Silymarin’s potent antioxidant properties neutralize free radicals and alleviate oxidative stress, safeguarding lung tissues from harm. In the context of sepsis-induced ALI, inflammation is a critical aggravating factor. Silymarin’s anti-inflammatory capabilities inhibit the activation of inflammatory cells and the release of inflammatory mediators, thereby diminishing inflammatory reactions and consequent lung injury.

Furthermore, silymarin enhances cellular defense mechanisms by boosting intracellular levels of glutathione, a significant antioxidant that shields cells from oxidative stress. It also prevents thrombosis by reducing the formation of blood clots in pulmonary vessels, further minimizing lung damage. Through its comprehensive antioxidant, anti-inflammatory, cytoprotective, and anticoagulant properties, silymarin stands as a promising agent in the therapeutic arsenal against sepsis-induced ALI [90].

### 3.15. Honokiol (***15***)

Honokiol (Table 2) is a natural compound from the genus *Magnolia* of the Magnoliaceae family, extracted from the bark and roots of the *Magnolia officinalis* tree. Renowned for its antioxidant, anti-inflammatory, antitumor, antimicrobial, and antidepressant properties, honokiol holds promise for therapeutic applications across various conditions, including neurological disorders, cardiovascular diseases, and infections [91].

During sepsis, honokiol markedly reduces the expression of inducible nitric oxide synthase and NF-κB in the lungs, showcasing its potent protective effects. Furthermore, it effectively mitigates pulmonary edema and pathological lung changes, significantly improving survival rates in septic mice. These findings underscore honokiol’s potential to inhibit lethal outcomes and ALI associated with sepsis, highlighting its therapeutic promise [92].

### 3.16. Resveratrol (***16***)

Resveratrol (Table 2), a natural polyphenol and a member of the stilbenoid class, is present in over 70 plant species, with a notable abundance in the skins of red grapes. It is also found in tea, berries, pomegranates, nuts, blueberries, and dark chocolate, each offering varying levels of resveratrol [93].

It has been found that sepsis-induced ALI is mainly associated with over-activation of the inflammatory response. Resveratrol can inhibit the inflammatory response by activating Sirt1, thereby reducing lung injury. Specifically, resveratrol can inhibit the overexpression of inflammatory mediators, for instance, matrix metalloproteinase 9 (MMP-9), IL-1β, IL-6, and iNOS, thereby reducing the extent of the inflammatory response. Moreover, resveratrol is crucial in diminishing the incidence of pulmonary edema, a critical ALI symptom and a common cause of mortality among patients, thus enhancing lung functionality. Overall, resveratrol’s action in treating sepsis-induced ALI involves dampening the inflammatory response and lessening pulmonary edema through Sirt1 activation [94].

In 2018, Gao’s study [95] found that resveratrol could attenuate sepsis-induced ALI and was associated with an increase in the expression of vascular endothelial growth factor (VEGF-B) correlation. By performing in vitro experiments in LPS-stimulated mouse alveolar macrophages (MH-S) cells, it was found that resveratrol administration inhibited LPS-stimulated TNF-α, IL-6, and IL-1β production. It was associated with inhibition of nuclear factor-κB, P38, and ERK signaling pathways. In addition, Resveratrol administration reduced apoptosis in MH-S cells by altering the BCL2-associated X protein (Bax)/B-Cell CLL/Lymphoma 2 (BCL2) balance and inhibiting LPS-induced autophagy. The inhibitory effects of resveratrol on both cytokine levels and apoptosis in alveolar macrophages were blocked by VEGF-B siRNA. Resveratrol administration modulated LPS-induced expression of C5aR and C5L2, revealing another mechanism for resveratrol’s anti-inflammatory and anti-apoptotic effects. These results suggest that resveratrol can protect against sepsis-induced ALI by activating the VEGF-B signaling pathway. There are several different pathways for resveratrol treatment of sepsis-induced ALI.

### 3.17. Curcumin (***17***)

Curcumin (Table 2), a natural compound extracted from turmeric, is the plant’s principal active ingredient and is widely grown in Asia. Renowned for its vibrant yellow hue, curcumin is a food seasoning and a valued component in herbal medicine. It exhibits many biological activities, including anti-inflammatory, antioxidant, antitumor, and wound-healing effects. Due to its extensive research backing, curcumin has significant medicinal potential [96].

In ARDS, curcumin is an antioxidant and a preventive agent against ALI triggered by various inhalants. It demonstrates a potent inhibitory action on pro-inflammatory cytokines, such as TNF-α, IL-1, IL-6, cyclooxygenase-2 (COX-2), and iNOS, and on transcription factors like NF-κB and activator protein-1 (AP-1). A study examining the impact of curcumin on sepsis-induced ALI, utilizing a rat model, investigated its effects on TGF-β1 expression. The findings revealed that curcumin treatment notably reduced the lung wet-to-dry weight ratio in rats with sepsis-induced ALI [97].

Overall, curcumin effectively mitigated the injury and inflammatory response in lung tissue, thereby preserving the typical structure of the lung. These outcomes imply that curcumin may deliver its therapeutic benefits in sepsis-induced ALI by targeting the TGF-β1/ mothers against the decapentaplegic homolog 3 (SMAD3) signaling pathway [97,98].

### 3.18. Glycyrrhizic Acid (GA, ***18***)

GA (Table 2) can exert its therapeutic effects on sepsis-induced acute lung injuries in several ways. GA treatment inhibited oxidative stress damage and apoptosis in lung tissues induced by ALI. Finally, GA treatment noticeably inhibited the activation of NF-κB, JNK, and P38 MAPK. These results suggest that GA protects against sepsis-induced ALI by inhibiting inflammatory response, oxidative stress injury, apoptosis, and inactivating NF-κB and MAPK signaling pathways. This provides a molecular basis for new medical treatments for sepsis-induced ALI [98].

GA can mitigate the effects of sepsis-induced acute lung injuries through multiple therapeutic mechanisms. The administration of GA has been shown to reduce oxidative stress and apoptosis in lung tissues affected by ALI. Significantly, GA treatment also markedly suppressed the activation of critical inflammatory signaling pathways, including NF-κB, JNK, and P38 MAPK. These findings indicate that GA is protective against sepsis-induced ALI by dampening the inflammatory response, mitigating oxidative stress and apoptosis, and deactivating the NF-κB and MAPK signaling pathways. Consequently, this underscores the potential of GA as a foundation for developing novel treatments for sepsis-induced ALI [99].

### 3.19. Bakuchiol (***19***)

Bakuchiol (Table 3) is a compound extracted from the fruit of *Psoralea corylifolia*, belonging to the class of terpenoids. It exhibits various pharmacological effects, including antioxidant, antimicrobial, anti-inflammatory, anti-aging, and estrogen-like properties [100]. Bakuchiol, conversely, can inhibit inflammatory responses and reduce the release of inflammatory mediators, such asTNF-α and IL-1β, thereby mitigating inflammatory damage in lung tissue. It may also alleviate oxidative stress by scavenging free radicals and enhancing antioxidant enzyme activity, thus protecting lung tissue from oxidative harm. Furthermore, bakuchiol can boost the expression of adhesion molecules (e.g., ICAM-1) in pulmonary vascular endothelial cells, consequently diminishing the infiltration and injury caused by inflammatory cells. It can also decrease the occurrence of apoptosis by modulating the expression of apoptosis-related proteins (e.g., Bcl-2 and Bax), protecting lung tissue from damage. Bakuchiol delivers its therapeutic effects on sepsis-induced ALI through various mechanisms, including anti-inflammatory and antioxidant activities and protecting pulmonary blood vessels and epithelial cells [101].

### 3.20. Terretonin (***20***)

Terretonin (Table 3), a natural product isolated from the fungus *Aspergillus terreus*, has been identified to exhibit various biological activities, including antioxidant, anti-inflammatory, and antimicrobial properties. Recent research suggests that Terretonin also holds promise in offering a protective effect against sepsis-induced ALI. It exerts its protective effects by modulating the SIRT1/Nrf2/NF-κBp65/NLRP3 signaling pathway. These findings propose that Terretonin may be a potential candidate for treating sepsis-induced ALI, indicating its therapeutic potential in addressing this condition [102].

### 3.21. Ginsenoside Rg1 (Rg1, ***21***)

Rg1 (Table 3), an active ingredient extracted from *Panax ginseng*, has demonstrated the ability to reduce inflammatory factors produced by inflammatory cells, such as IL-1β and IL-18, thereby exhibiting anti-inflammatory effects. It can also diminish the production of free radicals and shield cells from damage induced by oxidative stress. Additionally, Rg1 can decrease apoptosis in nerve cells, foster their survival and regeneration, and offer protection against brain damage and neurodegenerative diseases [103].

Rg1 employs a multiple approach to mitigate the effects of sepsis-induced ALI. Specifically, Ginsenoside Rg1 alleviates sepsis-induced ALI through various mechanisms, including reducing apoptosis, inhibiting the inflammatory response, and lessening lung tissue injury [104].

### 3.22. Astragaloside IV (AS-IV, ***22***)

AS-IV (Table 4), an active ingredient extracted from *Astragalus membranaceus*, is a triterpenoid saponin compound with a broad spectrum of applications in traditional Chinese medicine, particularly in treating cardiovascular diseases, digestive diseases and other conditions with high incidence and risk [105].

Specifically, AS-IV possesses an anti-inflammatory effect, inhibiting the onset of inflammatory reactions, a key mechanism in developing sepsis-induced ALI. It may reduce lung injury by suppressing the production of inflammatory factors and activating inflammatory cells. AS-IV has an antioxidant effect, scavenging free radicals and mitigating lung tissue damage caused by oxidative stress, another significant mechanism behind sepsis-induced ALI. Thus, AS-IV can protect lung tissue by counteracting oxidative stress. AS-IV therapeutically affects sepsis-induced ALI through anti-inflammatory, antioxidant, and cytoprotective actions. However, further research is required to elucidate its specific mechanisms and efficacy [106].

### 3.23. Aescin (***23***)

Aescin (Table 4), a natural compound extracted from the seeds of *Aesculus hippocastanum*, falls within the triterpenoid saponin class and is renowned for its broad spectrum of pharmacological properties, including anti-inflammatory, antioxidant, and antithrombotic activities [107,108].

Aescin effectively inhibits inflammatory responses and diminishes inflammatory mediators’ release. Its antioxidant capabilities enhance the activity of antioxidant enzymes, bolstering the cells’ antioxidant defenses. Furthermore, Aescin can suppress the activation of NLRP3 inflammasomes, thereby reducing inflammasome-mediated inflammatory responses. This contributes to a decrease in the release of inflammatory factors and lessens inflammatory damage in lung tissue. Consequently, Aescin delivers its therapeutic effects on sepsis-induced ALI through multiple mechanisms, such as anti-inflammatory actions, antioxidant effects, and the protection of the pulmonary vascular barrier. It alleviates inflammatory responses, mitigates oxidative stress, reduces pulmonary edema, and safeguards lung tissues from further damage [108,109].

### 3.24. Senegenin (***24***)

Senegenin (Table 4), an active compound extracted from the roots of *Polygonum multiflorum*, is recognized for its neuroprotective effects. It has been studied for its potential to enhance learning and memory capacity and to prevent postoperative cognitive dysfunction, marking it as a natural compound with considerable medicinal value [110,111].

In sepsis-induced ALI, senegenin demonstrates efficacy by reducing oxidative stress and inhibiting inflammation [112]. Studies in a rat model of sepsis revealed that senegenin treatment significantly lowered the lung’s wet/dry weight ratio and notably decreased the activity of myeloperoxidase (MPO), a marker for neutrophil activation. Further assessment of oxidative stress in lung tissue highlighted potential mechanisms for its protective effect against sepsis-induced ALI. Senegenin markedly reduced malondialdehyde (MDA) levels in lung tissue while increasing SOD activity and glutathione (GSH) content, indicating its role in mitigating oxidative stress in the lungs. In summary, senegenin treatment has been shown to attenuate sepsis-induced ALI, suggesting its potential as a therapeutic agent for treating this condition [113].

### 3.25. Betulinic Acid (BA, ***25***)

BA (Table 4), a natural product found abundantly in various plants such as birch bark, olive bark, and apple peel, exhibits a broad spectrum of biological activities, including anticancer, anti-inflammatory, antiviral, and antioxidant effects [114].

In a study [115], researchers utilized a mouse model to induce polymicrobial sepsis and administered BA as a pretreatment before surgery. The findings revealed that BA significantly reduced the levels of inflammatory cytokines, such as TNF-α, IL-1β, and IL-6. These results suggest that BA provides a therapeutic effect on sepsis-induced ALI by inhibiting the release of inflammatory cytokines and mitigating the inflammatory response. Consequently, BA may offer potential as an anti-inflammatory agent for treating ALI associated with sepsis [115,116].

### 3.26. Osthole (***26***)

Osthole (Table 5), a natural coumarin first extracted from the serpentine plant, boasts a wide array of pharmacological effects, including neuroprotection, osteogenesis, immunomodulation, anticancer, hepatoprotection, cardiovascular protection, and antimicrobial activity [117]. It is known for its capacity to inhibit inflammatory responses, mitigate oxidative stress, and enhance cognitive function. The diverse pharmacological impacts of osthole can be attributed to its regulatory effects on the levels of cyclic adenosine monophosphate (cAMP) and cyclic guanosine monophosphate (cGMP) [118].

Research has demonstrated osthole’s anti-inflammatory and antioxidant properties, highlighting its potential therapeutic benefits in treating sepsis-induced ALI (ALI). Osthole achieves its therapeutic effects by targeting and inhibiting the NF-κB signaling pathway. In research utilizing sepsis-induced ALI models, osthole demonstrated a decrease in the neutrophil count within alveolar lavage fluid, alongside a reduction in myeloperoxidase (MPO) activity, a key indicator of neutrophil activity in lung tissue. Furthermore, osthole effectively lowered the levels of inflammatory cytokines such as IL-6 and TNF-α in both alveolar lavage fluid and serum. Overall, osthole’s ability to inhibit the NF-κB signaling pathway and reduce inflammatory cell infiltration and cytokine secretion underscores its therapeutic potential in managing sepsis-induced ALI [119].

### 3.27. Songorine (***27***)

Songorine (Table 5), a diterpenoid alkaloid extracted from *Aconitum carmichaelii Debeaux*, exhibits a broad spectrum of pharmacological effects, including antiarrhythmic, central-nervous-system-stimulating, analgesic, and anti-inflammatory properties [120]. This C20-diterpene alkaloid, known for its anti-inflammatory capabilities and low toxicity, has shown promise in reducing sepsis-induced ALI through several mechanisms.

The therapeutic effect of songorine on sepsis-induced ALI is predominantly mediated by activating the PI3K/AKT/Nrf2 signaling pathway. Specifically, it has proven effective in reducing inflammatory cell infiltration and the release of inflammatory mediators in lung tissues, thus mitigating ALI. Songorine boosts levels of antioxidants such as SOD and GSH while diminishing the production of oxidative stress markers, thereby helping to maintain the oxidative-antioxidant balance and minimize oxidative-stress-induced damage to lung tissue. The activation of the PI3K/AKT/Nrf2 pathway by songorine plays a crucial role, as it fosters the antioxidant response and diminishes inflammation, enhancing anti-oxidative stress capabilities and reducing ALI [120,121].

In summary, songorine’s therapeutic effects on sepsis-induced ALI are achieved by suppressing inflammatory responses, mitigating oxidative stress, and activating the PI3K/AKT/Nrf2 signaling pathway. These actions work in concert to offer a protective effect on lung tissue [122].

### 3.28. Loganin (***28***)

Loganin (Table 5), a natural product classified within the terpenoid family, features a chemical structure enriched with functional groups such as glycosides and terpene rings, contributing to its good solubility in water [123]. This compound has shown promise in alleviating sepsis-induced ALI through its ability to modulate macrophage polarization and inhibit the activation of the NLRP3 inflammasome.

Loganin effectively reduces the release of M1-type macrophage-associated pro-inflammatory cytokines while inducing the activation of M2-type anti-inflammatory cytokines [124]. It inhibits NLRP3 inflammasome-mediated cysteine-requiring aspartate protease 1(caspase-1) activation and IL-1β secretion. Further in vitro research has validated that loganin can inhibit M1-type macrophage polarization and the activation of NLRP3 inflammasomes by blocking the ERK and NF-κB pathways. Consequently, the anti-inflammatory effects of loganin in sepsis-induced ALI are linked to its modulation of ERK and NF-κB pathway-mediated macrophage polarization and the inhibition of NLRP3 inflammasome activation [123,124].

### 3.29. Zerumbone (***29***)

Zerumbone (Table 5), a natural compound from the ginger family, has been the subject of extensive research due to its wide range of pharmacological activities, including anti-inflammatory, antioxidant, antitumor, antibacterial, and antidiabetic properties [125]. This makes zerumbone a promising candidate for natural drug development with significant potential for biomedical applications [126].

Zerumbone’s protective effect against sepsis-induced ALI is primarily attributed to its potent anti-inflammatory and antioxidant properties. It achieves these effects by inhibiting the NF-κB pathway and activating the heme oxygenase-1 (HO-1) pathway, which collectively reduces the infiltration of inflammatory cells and the concentration of inflammatory cytokines. The overactivation of NF-κB, commonly observed in sepsis, increases inflammation and tissue damage. Zerumbone intervenes by inhibiting NF-κB activation and reducing the production of inflammatory mediators, thereby mitigating the inflammatory response and subsequent injury. In summary, zerumbone’s ability to attenuate sepsis-induced ALI stems from its dual action of inhibiting the NF-κB pathway and activating the HO-1 pathway, underscoring its therapeutic potential through anti-inflammatory and antioxidant activities [127].

### 3.30. Syringaresinol (***30***)

Syringaresinol (Table 5), a natural lignan compound extracted from various plants such as spruce, pine, and Yunnan Baiyao, exhibits a range of biological activities, including antioxidant, anti-inflammatory, and antitumor effects [128,129].

Wang’s group [130] found that in mice treated with syringaresinol, there was a down-regulation in the protein expression levels of NLRP3, ASC, GSDMD, caspase-1 p20, and TLR4, along with reduced phosphorylation levels of NF-κB, ERK, JNK, and P38 in lung tissues. These findings indicate that syringaresinol can effectively prevent sepsis-induced inflammatory cell death in the lungs. Additionally, they observed that syringaresinol could inhibit LPS-induced apoptosis in an in vitro cellular assay.

In summary, syringaresinol exerts a protective effect against sepsis-induced ALI by inhibiting the activation of NLRP3 inflammasomes, potentially through the modulation of the estrogen receptor-β (ERβ) signaling pathway. These results suggest that syringaresinol holds promise as a potential therapeutic agent for treating sepsis-induced ALI.

### 3.31. Salidroside (***31***)

Salidroside (Table 5), primarily found in herbal medicines such as *Rhodiola rosea* and *Rhodiola crenulata* [131], is celebrated for its diverse pharmacological activities, including antioxidant, anti-inflammatory, antitumor, antidiabetic, and anti-cardiovascular effects. Recognized as one of the main active components of *Rhodiola rosea*, salidroside exhibits a broad spectrum of medicinal potential [132].

Salidroside offers therapeutic benefits in treating sepsis-induced ALI by engaging in distinct mechanisms at different stages of sepsis. In the early phase of sepsis, it attenuates the production of inflammatory factors such as TNF-α and IL-6 via the SIRT1-mediated NF-κB inhibition pathway. Progressing into the advanced stage of sepsis, Salidroside continues to confer protection against sepsis-induced ALI through the SIRT1-mediated HMGB1 nucleoplasmic translocation pathway. This dual-phase action suggests that Salidroside could be a promising therapeutic agent for addressing sepsis-induced ALI and potentially reducing mortality associated with sepsis. However, further research is essential to fully understand the intricate molecular mechanisms by which Salidroside operates in the context of sepsis treatment and to assess its viability as a novel strategy in clinical sepsis management [133].

### 3.32. Astaxanthin (***32***)

Astaxanthin (Table 5), a red fat-soluble pigment naturally occurring within the carotenoid group, is renowned for its wide array of pharmacological effects, including antioxidant, anti-inflammatory, antitumor, anti-aging, cardiovascular health protection, and immune system enhancement. It also offers eye protection, skin health improvement, and brain health promotion. The primary mechanisms through which Astaxanthin exerts its pharmacological effects involve scavenging free radicals, inhibiting inflammatory responses, and modulating cell signaling [134,135].

Research conducted by Cai [136] demonstrated that Astaxanthin significantly reduces LPS-induced infiltration of inflammatory cells in lung tissue. Additionally, it was found to decrease the serum levels of inflammatory cytokines, including IL-6 and TNF-α. Astaxanthin’s anti-inflammatory and antioxidant properties mitigate the sepsis-induced inflammatory response and oxidative stress, safeguarding lung tissue against damage. By repressing the activation of the MAPK/NF-κB signaling pathway, Astaxanthin curtails the production of inflammatory factors, further alleviating lung injury. In summary, Astaxanthin offers therapeutic benefits for sepsis-induced ALI through its anti-inflammatory and antioxidant properties, alongside the inhibition of the MAPK/NF-κB signaling pathway.

## 4. Discussion and Prospect

Sepsis is a systemic inflammatory condition triggered by pathogenic microorganisms infiltrating the body through various routes, affecting multiple systems and organs. The lungs are particularly vulnerable, and patients with severe infections are highly susceptible to ALI. The classic clinical symptoms of ALI include refractory hypoxemia and respiratory distress, with a morbidity and mortality rate of up to 40%. Despite extensive research into the mechanisms of sepsis-induced ALI, its exact pathological process remains elusive. Currently, there are no definitive cures for ALI, and the possibility of reversing its pathological process is a subject of debate. For sepsis-induced ALI, contemporary medicine primarily focuses on preventive, therapeutic strategies to mitigate and improve its pathological progression. Thus, developing targeted small-molecule drugs for ALI is of significant clinical importance, offering a solid foundation for its treatment.

The mechanism of ALI in sepsis involves various aspects, including inflammation response, oxidative stress, inflammatory cell infiltration, and vascular leakage. As shown by clinical research data, various biological signaling pathways play an influential role in regulating the process of sepsis-induced ALI, the most critical of which is the biological signaling pathway associated with the inflammatory pathway. If the inflammatory response is not controlled in time, it will become a more severe system failure. Clinical trials have shown that ALI or ARDS can be effectively treated with different strategies. Antibiotics or hemodialysis can effectively relieve ALI, while several naturally occurring small-molecule drugs can effectively treat ALI. Examples are AMPK inhibitors, PI3K δ/γ inhibitors, ROCK inhibitors, and many other drugs. In brief, these compounds, with their sepsis-induced infectious properties, hold great therapeutic promise for treating sepsis-induced ALI.

Following sepsis-induced ALI, certain medications pose the risk of triggering ARDS or exacerbating shock during treatment, such as the use of broad-spectrum antibiotics or glucocorticoids. These can introduce additional diseases, further straining the body’s organs and potentially leading to severe lung damage and failure. Therefore, it is crucial to prioritize lung function protection and minimize the risk of sepsis or ALI in our daily lives.

Beyond the compounds and natural products previously mentioned, gene therapy and drug-targeted systems are emerging as innovative strategies for treating sepsis-induced ALI. The exploration of therapeutic approaches for ALI associated with sepsis is progressing rapidly, with specific treatment guidelines expected to appear in the forthcoming years.

## Figures and Tables

**Table 1:** Sources, classifications, and structures of compounds **1**–**14**.

No.	Natural Drugs	Sources	Classifications	Structures
**1**	Dihydromyricetin	*Ampelopsis grossedentata*	Flavonoid	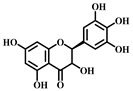
**2**	Myricetin	*Myrica rubra* L.	Flavonoid	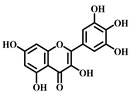
**3**	Luteolin	*Brassica oleracea* *Piper guineense*	Flavonoid	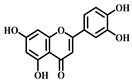
**4**	Quercetin	*Quercus acutissima*	Flavonoid	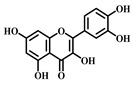
**5**	Baicalein	*Scutellaria baicalensis-* Georgi	Flavonoid	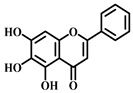
**6**	Kaempferol	*Kaempferia galanga* L.	Flavonoid	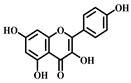
**7**	Cardamonin	*Elettaria cardamomum* L.	Flavonoid	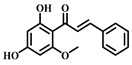
**8**	Isoliquiritigenin	*Glycyrrhiza glabra* *Mongolian glycyrrhiza*	Flavonoid	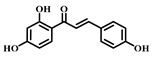
**9**	Afzelechin	*Bergenia ligulata*	Flavonoid	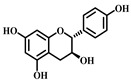
**10**	Calycosin	*Astragalus penduliflorus*	Flavonoid	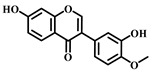
**11**	Astilbin	*Astilbe chinensis*	Flavonoid	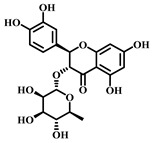
**12**	Gossypin	*Hibiscus syriacus* L.	Flavonoid	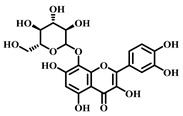
**13**	Isoorientin	*Polygonum aviculare* L.	Flavonoid	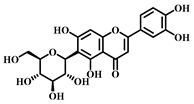
**14**	Silymarin	*Silybum marianum* L.	Flavonoid and Lignan	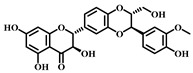

**Table 2:** Sources, classifications, and structures of compounds **15**–**18**.

No.	Natural Drugs	Sources	Classifications	Structures
**15**	Honokiol	*Magnolia grandiflora* *Magnolia dealbata*	Polyphenol	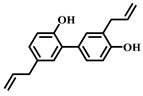
**16**	Resveratrol	*Vitis Ampelopsis*	Polyphenol	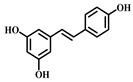
**17**	Curcumin	*Curcuma aromatica Salisb* *Curcuma longa* L.	Natural phenol	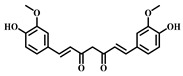
**18**	Glycyrrhizic Acid	*Glycyrrhiza uralensis Fisch*	Terpenoid	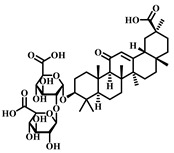

**Table 3:** Sources, classifications, and structures of compounds **19**–**21**.

No.	Natural Drugs	Sources	Classifications	Structures
**19**	Bakuchio	*Psoralea corylifolia* L.	Terpene	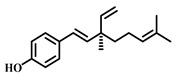
**20**	Terretonin	*Aspergillus terreus*	Terpene	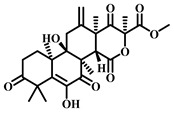
**21**	Ginsenoside Rg1	*Panax ginseng*	Triterpenoid saponin	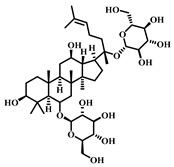

**Table 4:** Sources, classifications, and structures of compounds **22**–**25**.

No.	Natural Drugs	Sources	Classifications	Structures
**22**	Astragaloside IV	*Astragalus membranaceus*	Triterpenoid saponin	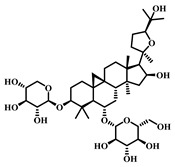
**23**	Aescin	*Aesculus hippocastanum*	Triterpenoid saponin	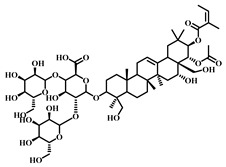
**24**	Senegenin	*Polygala tenuifolia*	Saponin	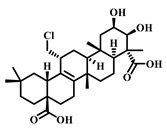
**25**	Betulinic Acid	*Betula pubescens*	Pentacyclic triterpenoid	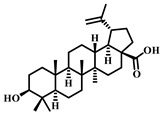

**Table 5:** Sources, classifications, and structures of compounds **26**–**32**.

No.	Natural Drugs	Sources	Classifications	Structures
**26**	Osthole	*Cnidium monnieri*	Coumarin	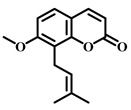
**27**	Songorine	*Aconitum carmichaelii Debeaux*	Alkaloid	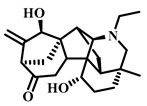
**28**	Loganin	*Cephalosporium aphidicola*	Iridoid	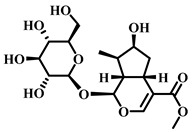
**29**	Zerumbone	*Zingiber zerumbetSmith*	Terpene	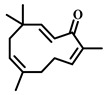
**30**	Syringaresinol	*Sargentodoxa cuneata*	Lignin	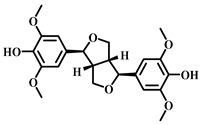
**31**	Salidroside	*Rhodiola rosea* *Rhodiola jadea*	Glycoside	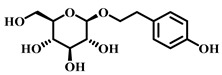
**32**	Astaxanthin	*Haematococcus pluvialis*	Terpene	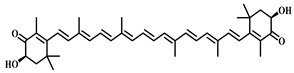

## Data Availability

Not applicable.

## Abbreviations

ALI	Acute lung injury
SIRS	Systemic inflammatory response syndrome
Sepsis-3	The publication of the third international consensus definition of sepsis and septic shock
qSOFA	Quick SOFA
ARDS	Acute respiratory distress syndrome
TNF-α	Tumor necrosis factor-α
IL-1β	Interleukin-1β
IL-6	Interleukin-6
NF-κB	Nuclear factor-κB
PI3K	Phosphatidylinositol-3 kinase
AKT	Protein kinase B
MNK	Mitogen-activated protein kinase interacting kinases
ErK	Extracellular signal-regulated kinase
LPS	Lipopolysaccharide
PI3Kδ/γ	Phosphoinositide 3-kinase delta/gamma pathway
ROCK	Rho-associated protein kinase
Tie2	Tyrosine kinase with immunoglobulin and epidermal growth factor homology domains 2
MD2	Myeloid differentiation factor 2
TLR4	Toll-like receptor 4
TLRs	Toll-like receptors
TNF	Tumor necrosis factor
ILs	Interleukins
ROS	Reactive oxygen species
PAMPs	Pathogen-associated molecular patterns
DAMPs	Damage-associated molecular patterns
CLP	Cecal ligation and puncture
WT	Wild-type
MyD88	Myeloid differentiation primary response 88
IL-1R	Interleukin-1 receptor
IRAK	IL-1R-associated kinase
NOX	NADPH oxidase
O^2−^	Superoxide anion
AMPK	Adenosine monophosphate-activated protein kinase
ECM1	Extracellular matrix protein 1
STAT5	Signal transducer and activator of transcription 5
NLRP3	Nucleotide-binding oligomerization domain-like receptor protein 3
TAN	Tangeretin
PLK1	Polo-like kinase 1
DRP1	Dynamin-related protein 1
STAT3	Signal transducer and activator of transcription 3
GSDM	Gasdermin D
ERRα	Estrogen-related receptor alpha
Pla2g2a	Phospholipase A2 type IIA
EGFR	Epidermal growth factor receptor
MUC1	Mucin 1
miR-199a	microRNA 199a
DHM	Dihydromyricetin
Nrf2	Nuclear factor erythroid 2-related factor 2
HO-1	Heme oxygen-ase-1
ICAM	Intercellular adhesion molecule
iNOS	Inducible nitric oxide synthase
SOD	Superoxide dismutase
CAT	Catalase
NO	Nitric oxide
ISL	Isoliquiritigenin
MDA	Malondialdehyde
GSH	Glutathione
MIF	Macrophage inhibitory factor
JAK2	Janus kinase 2
GA	Glycyrrhizic acid
Rg1	Ginsenoside Rg1
AS-IV	Astragaloside IV
BA	Betulinic Acid
cAMP	Cyclic adenosine monophosphate
cGMP	Cyclic guanosine monophosphate
ER-β	Estrogen receptor-β
MAPKs	Mitogen-activated protein kinases
EPCR	Endothelial protein C receptor
MPO	Myeloperoxidase
JNK	c-JunN-terminalkinases
mTOR	Mammaliantarget of rapamycin
IKK	Inhibitor of kappa B kinase
VEGF	Vascular endothelial growth factor
AP-1	Activator protein-1
PGC-1α	Peroxisome proliferator-activated receptor-γ coactivator 1-α
CaMKII	Ca^2+^/calmodulin-dependent protein kinase II
MLCK	Myocin light chain kinase
HMGB1	High mobility group protein 1
MMP-9	Matrix metalloproteinase 9
MH-S	Mouse alveolar macrophages
COX-2	Cyclooxygenase-2
SMAD3	Mothers against decapentaplegic homolog 3
Bcl-2	B-Cell CLL/Lymphoma 2
Bax	BCL2-associated X protein
Caspase-1	Cysteine-requiring aspartate protease-1
